# ‘She mustn’t be the mother alone’: motivators, barriers, and recommendations for men’s engagement with reproductive research in South Africa

**DOI:** 10.1093/heapro/daaf207

**Published:** 2025-12-02

**Authors:** Caitlin V Gardiner, Tshepang Headman, Larske M Soepnel, Jackson Mabasa, Sonja Klingberg, Molebogeng Motlhatlhedi, Michelle Pentecost, Catherine E Draper, Lukhanyo H Nyati

**Affiliations:** Department of Global Health and Social Medicine, King’s College London, 40 Aldwych, London, WC2B 4BG, United Kingdom; SAMRC/Wits Developmental Pathways for Health Research Unit, Department of Paediatrics, School of Clinical Medicine, Faculty of Health Sciences, University of the Witwatersrand, Private Bag X3 Wits, 2050, Johannesburg, South Africa; SAMRC/Wits Developmental Pathways for Health Research Unit, Department of Paediatrics, School of Clinical Medicine, Faculty of Health Sciences, University of the Witwatersrand, Private Bag X3 Wits, 2050, Johannesburg, South Africa; SAMRC/Wits Developmental Pathways for Health Research Unit, Department of Paediatrics, School of Clinical Medicine, Faculty of Health Sciences, University of the Witwatersrand, Private Bag X3 Wits, 2050, Johannesburg, South Africa; Division of Epidemiology and Biostatistics, School of Public Health, University of Cape Town, Observatory, 7925, Cape Town, South Africa; Julius Center for Health Sciences and Primary Care, University Medical Center, Huispost nr. STR 6.131, P.O. Box 85500, 3508 GA, Utrecht, The Netherlands; SAMRC/Wits Developmental Pathways for Health Research Unit, Department of Paediatrics, School of Clinical Medicine, Faculty of Health Sciences, University of the Witwatersrand, Private Bag X3 Wits, 2050, Johannesburg, South Africa; SAMRC/Wits Developmental Pathways for Health Research Unit, Department of Paediatrics, School of Clinical Medicine, Faculty of Health Sciences, University of the Witwatersrand, Private Bag X3 Wits, 2050, Johannesburg, South Africa; SAMRC/Wits Developmental Pathways for Health Research Unit, Department of Paediatrics, School of Clinical Medicine, Faculty of Health Sciences, University of the Witwatersrand, Private Bag X3 Wits, 2050, Johannesburg, South Africa; Department of Global Health and Social Medicine, King’s College London, 40 Aldwych, London, WC2B 4BG, United Kingdom; SAMRC/Wits Developmental Pathways for Health Research Unit, Department of Paediatrics, School of Clinical Medicine, Faculty of Health Sciences, University of the Witwatersrand, Private Bag X3 Wits, 2050, Johannesburg, South Africa; SAMRC/Wits Developmental Pathways for Health Research Unit, Department of Paediatrics, School of Clinical Medicine, Faculty of Health Sciences, University of the Witwatersrand, Private Bag X3 Wits, 2050, Johannesburg, South Africa; SAMRC/Wits Developmental Pathways for Health Research Unit, Department of Paediatrics, School of Clinical Medicine, Faculty of Health Sciences, University of the Witwatersrand, Private Bag X3 Wits, 2050, Johannesburg, South Africa; Interprofessional Education Unit, Faculty of Community and Health Sciences, University of the Western Cape, Private Bag X17, Bellville, 7535, Cape Town, South Africa

**Keywords:** research engagement, reproductive and lifecourse research, men in research, gender inequity, promoting gender justice

## Abstract

There is a notable lack of engagement in reproductive and lifecourse research with and from men, with a disproportionate focus on women. While studies investigating the lack of men’s engagement with *services* exist, there is little evidence on their engagement with reproductive and lifecourse *research*. This article qualitatively examines men’s engagement in such research in the *Bukhali* trial in South Africa. Single, in-depth interviews were conducted with 19 male partners of *Bukhali* participants, and data were thematically analysed. Results were grouped into three domains: (i) motivators for engagement, (ii) barriers to engagement, and (iii) recommendations to improve engagement. Motivators to engage included partner support, desiring to learn and gain knowledge, and taking the opportunity to access the same perceived trial benefits as their partner. Barriers included having misconceptions around the research, fear and mistrust of hospitals and illness, gendered ideas around ‘women’s health issues’, hesitance around sharing personal details, and lack of time and perceived (financial) benefits. To increase engagement, male partners recommended increasing awareness and creating safe research spaces for men. Each theme was inflected with male partners’ sociocultural and contextual realities and provided valuable insights into obstacles and opportunities for improving men’s engagement in reproductive research.

Contribution to Health Promotion

- Reproductive and lifecourse research overwhelmingly focuses on and engages women.
- Men’s health cannot be effectively promoted without engaging men in reproductive and lifecourse research.
- Building on scant prior research, this paper provides crucial insights directly from men about why they do or do not engage with reproductive research and their recommendations for improvement.
- Gaining men’s perspectives is crucial for designing health research and health promotion practices that are effective and equitable.

## INTRODUCTION

The lack of engagement with men regarding their own and their partner’s reproductive health has long been noted to be an obstacle to gender equity and justice (United Nations Department of Economic and Social Affairs 1995). Men’s engagement here refers to *all* the ways that men relate to and participate in reproductive behaviours, rights, health problems, research, and programmes—for themselves, their partners, and for parenting their children (Ibid.). In the context of research, specifically reproductive and lifecourse related, engaging men can build the evidence base and promote awareness about the paternal contribution to children’s and transgenerational health and enhance fathers’ involvement in child-rearing.

While the evidence base predominantly focuses on women, there is increasing evidence suggesting the enduring effects of men’s lifecourse behaviour and health, both for others and themselves. In the preconception period, men’s health can significantly affect the health trajectories of future children (Frey *et al*. 2008, Stuppia *et al*. 2015, Sharma *et al*. 2016). During pregnancy, male partner involvement can have positive effects for the mother and foetus through mitigating maternal gestational stress and later postnatal depression (Mezulis *et al*. 2004, Yargawa and Leonardi-Bee 2015, Jackson 2017, Drysdale *et al*. 2021). Paternal behaviours may also influence young children’s ongoing development (Makusha *et al*. 2019), e.g. paternal interaction during early childhood predicted preschoolers’ higher social competence and lower aggression (Feldman *et al*. 2013). Finally, evidence suggests that active paternal engagement positively affects men’s own physical and psychological lifecourse health (Bartlett 2004, Garfield *et al*. 2006, Kotelchuck and Lu 2017, Nelson-Coffey *et al*. 2019, Kotelchuck 2022). Thus, promoting men’s engagement in reproductive and lifecourse research, from preconception to early childhood, provides an opportunity to improve family planning and pregnancy results, promote the health of men and women, better prepare fathers, and positively impact child development (Frey *et al*. 2008).

Despite the increasing evidence suggesting the importance of men’s roles in their own and others’ lifecourse health, the failure to effectively engage men in research hampers further knowledge advancement (Culley *et al*. 2013, Davison *et al*. 2016, Soubry 2018b, Grace *et al*. 2019). This failure has frequently been framed as unwillingness of men to participate, eliding that most reproductive research does not recruit or include men (Saewyc 2012, Almeling and Waggoner 2013, Culley *et al*. 2013, Soubry 2018a, Grace *et al*. 2019, Mohr and Almeling 2020). Not only does this exclusion inhibit men’s health; it also has important sociological implications by re-enforcing unequal gender norms and reproducing notions that the responsibility for health, children, and future generations lies predominantly with women (Chiapperino and Panese 2018, Pentecost *et al*. 2018, Mello *et al*. 2019, Valdez 2021). This can then further dissuade men from participating in reproductive research (Grace *et al*. 2019) and reinforce the mothers’ tendency to gatekeep child-related activities, including research (Makusha and Richter 2016). Men’s research engagement is important not only to promote and normalize men’s involvement in child-bearing and child-rearing responsibilities but also to promote gender equality.

In South Africa (SA), relational dynamics intersect with an array of complex social, structural, and cultural factors that obstruct men’s involvement in reproductive and lifecourse research. This includes the socially disruptive legacies of apartheid and the human immunodeficiency virus (HIV) that continue to affect family dynamics and gendered relations (Makusha and Richter 2014, Manderson *et al*. 2016, Mkhwanazi and Manderson 2020). For example, only 35.7% of children cohabit with biological fathers (Stats 2022), which is rooted.in apartheid’s segregation laws and enduring socioeconomic conditions with resulting migrant labour practices for Black men. Importantly, however, not cohabiting does not equate to being uninvolved or unsupportive—this is a typically narrow and Eurocentric assumption that ignores more complex sociocultural relations (Makusha and Richter 2014, Morrell *et al*. 2016, Makusha and Van Den Berg 2018, Van den Berg *et al.* 2021, Bitalo *et al*. 2024). Nevertheless, limited paternal presence may represent a further obstacle to engaging men in reproductive research.

Additionally, the widespread effects of HIV have indelibly disrupted South African family and gender dynamics. The focus of HIV health promotion and interventions, however, has only reinforced gender inequity in reproductive health and research. This is because they have tended to focus heavily on women and children (Ma 2007, Ramjee *et al*. 2007, Montgomery *et al*. 2008, Venables and Stadler 2012), with a prominent example being the Preventing Mother to Child Transmission Initiative (PMTCT) (Nkomo 2015). As such, ‘men play little or no role in mitigating the consequences of HIV in affected families’ (Hosegood *et al*. 2016, p. N47). Additional documented factors obstructing South African men’s engagement with HIV care involve institutional and social barriers, including lack of ‘male-friendly’ HIV services, clinic factors such as opening hours and queues, stigma and privacy, and ideas around gender and masculinity (Sileo *et al*. 2018, Adeagbo *et al*. 2019, Adeagbo and Naidoo 2021). This is on top of obstacles to men’s general reproductive health engagement, including unemployment and poverty, negative health care worker attitudes, and lack of awareness and information (Mohlala *et al*. 2012, Nesane *et al*. 2016, Drysdale *et al*. 2021, Roudsari *et al*. 2023).

In 2020, in recognition of South African men’s low engagement with and by reproductive and health agendas generally, the National Department of Health South Africa (2020) implemented the South African National Integrated Men’s Health Strategy 2020–25. This strategy aims to ‘provide a comprehensive and integrated package of care for men and boys across the life course’ (Ibid, p. 3), which includes, but is not limited to, men’s sexual and reproductive health. Importantly, the strategy is committed to ‘Build[ing] the evidence base for improving men’s health’ (Ibid, p. 3), meaning men must be engaged in health *research*. As yet, however, the momentum behind reproductive research still focuses on women.

The evidence exploring men’s perspectives on engaging with reproduction-related lifecourse *research*, versus *services*, is sparse. Health services and research are distinct, and a better understanding of men’s perspectives specifically around research is crucial to gaining insights into how their engagement can be improved to build the evidence base. Drawing on qualitative work with the *Bukhali* trial in Soweto, Johannesburg, this study asks the question: what are the motivators, barriers, and suggested improvements for men’s engagement with reproductive and lifecourse health research in SA?

## METHODS

### Setting and participants:

*Bukhali* is the South African cohort of the Healthy Life Trajectories Initiative (HeLTI), which is an international research consortium investigating the longitudinal effects of behaviours and complex interventions primarily on intergenerational obesity (Norris *et al*. 2022, Draper *et al*. 2023b). It aims to generate evidence to promote healthier lifecourse trajectories for women and their children, from preconception to early childhood. Other cohort countries include Canada, China, and India. *Bukhali* launched in 2019 and recruited 6360 young women aged 18–28 and a subset of their male partners (Norris *et al*. 2022). It is the first intervention trial in SA to include a preconception component. *Bukhali* is based in Soweto—a Johannesburg township established under apartheid for Black urban inhabitants, characterized by enduring insecurity, poverty, and adversity (Bosire *et al*. 2021, Draper *et al*. 2023a).

This study formed part of *Bukhali’s* process evaluation that uses a mixed-methods approach to investigate the study context, trial implementation, and mechanisms of impact (Draper *et al*. 2023b). A subset of pregnant participants’ male partners (‘fathers’) were included in the process evaluation to gain insights into developing strategies that will promote men’s involvement during pregnancy and child-rearing (Draper *et al*. 2023a). In this article, we use data collected directly from these men to answer a research question speaking specifically to men’s engagement in reproductive research. This allowed us to develop relevant recommendations by foregrounding the opinions and priorities of those we wish to engage—South African men. This same process evaluation dataset was also used to answer a different question about relationship dynamics and perceptions of fatherhood; these are published elsewhere (Draper *et al*. 2023a).

Fathers were only contacted with prior *Bukhali* female participants’ agreement. This necessary safeguarding of the women resulted in unavoidable bias. The top three reasons for women’s refusal to contact the father were relationship breakdown following the pregnancy (26%), father’s lack of interest (20%), and father relocation (16%). If female participants agreed, however, fathers were recruited for biopsychosocial measurements and biological sample collection. From this invited group, 44 fathers were additionally recruited for in-depth interviews between 11 May 2022 and 31 September 2023. Of those invited, 27 agreed to participate—reasons for refusal are not known. Twenty-one fathers attended the interview, with a final sample of 19 interviews (due to the unfortunate loss of two interviews resulting from damaged recordings). Here, further unavoidable bias was introduced as only fathers already inclined and able to participate could provide data on men’s research engagement. The data thus only reflects the perceptions of men who were already more supportive and engaged.

### Data collection

Single in-depth interviews were conducted at the Developmental Pathways for Health Research Unit (DPHRU) between April and July 2022. A semistructured interview guide was developed by L.H.N., C.E.D., M.M., J.M., T.H., S.K., and M.P., integrating knowledge from the literature and local contextual experience in SA and Soweto. Questions explored the relationship between the father and the *Bukhali* participant, the fathers’ experiences of the pregnancy and trial, and their thoughts on what could improve men’s trial engagement. The trained interviewers and note-takers, T.H. and J.M., were male with fluency in English, and local languages interviews were conducted in English, isiZulu, Setswana, and Sesotho according to participant preference and lasted between 20 and 78 minutes (average of 37 minutes). Interviews were audio-recorded and transcribed *verbatim*, and translated into English where necessary by *Bukhali’s* chosen accredited translation service.

### Data analysis

In analysing our data, we predominantly used codebook thematic analysis similar to the template analysis (King 1998, Brooks *et al*. 2015). This flexible approach allowed us to develop *a priori* themes (namely motivators, barriers, and recommendations around men’s engagement) while remaining open to inductively generating themes and subthemes from the data. An initial codebook with themes and subthemes was developed with a few interview transcripts and then applied to the larger dataset where it was continuously and iteratively developed. In this research, our themes remained predetermined, while our subthemes were inductively generated. M.M. did initial data familiarization and codebook formulation. C.E.D. then reviewed the refined themes, and further theme and subtheme consolidation was done by M.M., C.E.D., L.M.S., C.V.G., L.H.N., T.H., and J.M., which enhanced analytic reliability. We further enhanced reliability by remaining reflexive about our researcher positionalities—rather than viewing these as an avenue for potential bias, however, we recognized the value that our subjectivities, especially combined, brought to the research as outlined by Braun and Clarke (2019). We also used Braun and Clarke’s (2020) tool for evaluating thematic analysis to ensure quality in our research reporting.

### Ethical considerations

This research is compliant with the ethical principles set out in the World Medical Association Declaration of Helsinki of 1975, as revised in 2008 (http://www.wma.net/en/30publications/10policies/b3/17c.pdf). Ethical approval for the #*Bukhali* trial was granted by the University of the Witwatersrand (M1811111, approved 20 February 2019; extension M240162, approved 05 February 2024). For this process evaluation study with fathers, ethical approval was granted by the University of the Witwatersrand (M190449, approved 26 April 2019, updated on 18 May 2022). It also forms part of C.V.G.’s PhD work, with ethical approval granted from King’s College London (LRS/DP-22/23-29682, approved 05 September 2022). Prospective participants were electronically provided with an information and consent document and given a minimum of 24 hours of deliberation and enquiry time. Participants provided voluntary written informed consent prior to interviews. Participants could withdraw consent without reason until 1 month following their own interview; subsequently, individuals’ data became inseparable from the whole. Participants consented to deidentified data access limited to the research team and the low risk of identification through quotes. Data protection and confidentiality will be maintained according to the South African Protection of Personal Information Act (POPIA) 4 of 2013 and the UK General Data Protection Regulation (GDPR UK).

### Positionality

We are a highly interdisciplinary team of researchers, with backgrounds spanning the social and medical sciences. Our positions range from master’s student to professor and principal investigator, and most of us are South African or have been in SA for at least 5 years. T.H., J.M., and L.H.N. are South African Black men, and M.M. is a South African Black woman, who grew up in similar circumstances as the participants. L.H.N. has a background in nutritional and lifecourse epidemiology. J.M., T.H., and M.M. are experienced field researchers who have intimate knowledge of Soweto. This research could not have occurred effectively without their epistemic vantages, and they played integral roles particularly in participant recruitment, data collection and fieldwork, data collation and curation, and data analysis. C.V.G., C.E.D., and M.P. are English-speaking South African women, and L.S. and S.K. are White European women, all with varying backgrounds in biomedicine. Additionally, C.V.G. and M.P. have dual training in bioethics and anthropology, respectively, and C.E.D., who oversees *Bukhali’s* intervention, control, and process evaluation components, is experienced in psychology, public health, and qualitative research methods. Reflexivity about our varying ontological and epistemic positions allowed us to work collaboratively and produce a cohesive piece of research that is locally representative and relevant.

## RESULTS

Our results are derived from interviews with 19 fathers from Soweto. Table 1 shows the fathers’ demographics.

**Table 1.** Fathers’ demographics.

Variable		*N* (%)
Age	21–30	5 (26.3)
	31–40	11 (57.9)
	41–50	3 (15.8)
Highest level of education	Grade 10	1 (5.3)
	Grade 11	5 (26.3)
	High school	8 (42.1)
	High school + certificate/course	3 (15.8)
	Diploma	2 (10.5)
Employment status	Employed	12 (63.2)
	Unemployed	7 (36.8)
Cohabiting with female *Bukhali* participant?	Yes	9 (47.4)
	No	10 (52.6)

Three overarching domains were constructed from the data regarding men’s engagement in reproductive research: (1) Motivators for fathers’ engagement, (2) Barriers for fathers’ engagement, and (3) Fathers’ recommendations for improving engagement (Table 2).

**Table 2.** Overview of themes.

Domain	Themes
1. Motivators for fathers’ engagement	Partner support
	The desire to learn
	Perceiving trial benefits through partner
2. Barriers for fathers’ engagement	Lack of understanding and misconceptions around health research
	Fear of hospital and illness
	Reluctance to engage with ‘women’s health issues’
	Resistance to sharing personal matters
	Lack of time and (financial) benefits
3. Fathers’ recommendations for improving engagement	Increasing awareness
	Creating a safe space

### Motivators for father’s engagement

#### Partner support

One of the reasons fathers described for wanting to be involved in *Bukhali* was as a way to support their partners during (and following) their pregnancy. Some fathers described their involvement in the trial as a natural result of their dedication to their partners by wanting a better understanding of their partner’s trial experience. In addition, some fathers described their participation as a way of actively showing their commitment to their partner and the pregnancy, especially considering the trial’s importance to female partners. Some fathers also described initially attending the study to show support for their partner, but subsequently becoming interested in the trial themselves.

I believe that it’s a way of supporting [female partner], doing this. Me doing it shows that I want better things for us and the baby; so in a way I’m doing it to encourage her as well. (IDI15)

Because I love her, and I wanted to be supportive of her, because we support each other in every little thing we do. (IDI3)

She told me that there’s this study and I said, “You know what, let me support her”, because if I don’t support her it’s going to end up badly and we’ll blame each other, so I said, “No, let me go and see for myself’, and then I became interested in how they do things here”. (IDI8)

#### The desire to learn

Fathers described their desire to learn new things through the trial as another main motivator for engagement, including how to become a ‘better father’. Fathers perceived the trials being able to provide education in the form of direct advice, and also a better understanding of their female partners’ needs during pregnancy, with an acknowledgement of previous relationship issues and lack of support provided.

Maybe your advice will work on me in increasing, what can I say, just me becoming a better father to my child and partner. I can maybe be a better person. (IDI17)

I’m interested because maybe I’m the one who is missing out, cause I also want to be on the right track, it mustn’t just be her … she mustn’t be the mother alone, as if she’s the only parent for the child; so I also wanna be on the same lane…maybe I would go there and get new advice or maybe I’m also the problem…maybe there’s something that I’m not doing right. (IDI11)

The desire to learn also seemed to stem from not wanting to miss out on the knowledge they witnessed their partners gaining from the trial (also see Theme 1.3), including around pregnancy and health. This interest extended to the health literacy resources provided by the trial.

Sometimes when she went out, I wouldn’t show her that I also read it [Bukhali resource book], I’d steal it… How to wash a baby, how to change diapers, a lot of things which I now know how to do. (IDI3)

Fathers also described their interest in learning more about their own health status from the data collection appointment.

And then she [research nurse] was measuring my heart before I started exercising and then she measured my heartbeat after the exercise. And then she was explaining to me the difference… so that was exciting for me. (IDI6)

You can get checked in terms of what is not going well in your body and everything; that made me happy and I was like “let me go and check if there’s anything wrong with me”. (IDI13)

#### Perceiving trial benefits through partner

Fathers noted numerous benefits that their female partners received from the trial, and their resulting positive regard for the trial seemed to make fathers more likely to participate themselves. The noted benefits for female partners included knowledge gained about pregnancy and motherhood, transportation reimbursements and health screenings, and receiving multi-micronutrient supplements. Furthermore, fathers’ general impression that their partners were well cared for seemed to increase their motivation to participate.

What made me happy, they used to visit her giving her pills during the pregnancy every month. That made me interested…I was interested that she was taken care of. (IDI10)

I was like, “Oh, thank you for telling me about the study’, because I felt I needed something like this to talk more about the experience, because the girl gets the most experience, you understand. So, what you had to do is buy nappies, buy baby wipes, gripe water, whatever; and then that is it, no, that’s not enough”. (IDI10)

The trial-based pregnancy ultrasound provided to female *Bukhali* participants, and the option for the fathers to be present for this, was also described as a benefit that brought the fathers closer to their partner and improved their opinion of the trial. Furthermore, the trial benefits seemed to be clearer to the fathers when they were able to accompany their partners to one of their trial sessions.

Until the chance I got when she came to the study, that’s when I started being with her for the sonar and what not, that’s when I started being close to her. (IDI1)

But what I liked is that it was the first time she did the scan; so I thought “oh so this thing works, this thing is legit”. (IDI3)

### Barriers to fathers’ engagement

#### Misconceptions and lack of information about the research

Uncertainty about what participation entails and misconceptions about research studies were described as significant reasons why men might decline participating. Lack of familiarity with the research unit fuelled mistrust and fear that the invitation to participate could be a scam.

Some people if they hear about the DPHRU they’re thinking of something else. They are thinking maybe it is a scam or it is something where they want to steal you, they want to take your phone or something. (IDI4)

In addition, the word research ‘study’ was reported to create the impression of (unqualified) students practicing and conducting trial procedures, with a perceived lack of professionalism and mistakes. This reflects an unintended epistemic mismatch, where the use of the word ‘study’ is not accompanied by contextually translated information for participants explaining what a research study actually entails.

When they say “study” you have this thing that whoever is going to do whatever it’s not going to be professional, they are still being taught…. that was my mind-set, these people are still students, they are not yet professional so there might be a mistake that happens because this person is probably still learning what they’re doing. (IDI1)

The variable degree to which female partners shared information about their participation in the trial may have contributed to this misunderstanding and lack of information amongst fathers.

We don’t discuss, there I’ll be honest, if she goes, she goes, and when she comes back she tells me that “I’m back, I’m done”, they checked what they checked; then we are done; I won’t ask her questions about but how do you find the whole thing, how does it help you and all that; no, I will be lying, I don’t question her. (IDI1)

#### Fear of hospital and illness

Fear of attending a hospital, particularly this specific public hospital, was another reason why fathers would choose to avoid engaging in health research. One element to this was fear of malpractice at such hospitals, which appears to reflect a deep mistrust of hospital-based biomedicine and practitioners amongst participants.

At first, I had a doubt about it, especially when someone says at Bara [DPHRU location]; the only thing that you think of at Bara, they are going to kill you now … … at Bara you should know when you get admitted they want your organs to give to other people. (IDI7)

Others are scared, like, hospitals do so, so, and so, you see. (IDI19)

The reluctance to attend hospitals was also due to fear of being confronted with a diagnosis and its consequences. The fathers mentioned that men may partake in high-risk health behaviours that amplify a fear of the health screenings conducted at the research study, particularly around HIV. The fathers implied that some men prefer ignorance over knowledge of a potential diagnosis and the associated stigma.

Guys are afraid of the hospital because guys get up to a lot of things that if you go to the hospital those things may emerge which you didn’t even know you had those things…We have this concept of it’s better that I don’t know, than to know. (IDI 1)

They check blood and stuff, so maybe others might be scared to find out things that they don’t think they’ll find out… Especially when it comes to testing HIV and things like that. (IDI15)

For them they see a hospital as a place of death, a place for weak people, you know that belief that men are not weak and all that; so they have that mind-set of “how will people look at me if they see me coming out of the hospital, people will start thinking I’m sick”. (IDI13)

#### Reluctance to engage with ‘women’s health issues

Another barrier to engagement was the impression that the research study focused on *women’s* health issues only. Father’s implied reluctance to get involved in what they considered predominantly ‘women’s issues’. This was out of a lack of interest, the feeling that it is private for women, or that they as men did not know enough about pregnancy and child-rearing to be able to participate. This last reason also suggests that participants may view research participation as a test of their knowledge in the area of research.

I know there is a study she went to for the vagina, then there is a study for pregnancy, I think it is the same study. And then there is a study after giving birth. But, in terms of the specifics, in terms of what is going on, I honestly don’t know. And I wouldn’t ask her, I want to ask her, but I feel like maybe private something, she is not supposed to tell me. (IDI9)

The reason why other guys refuse to come here is because they don’t know how they are going to answer the questions when asked, because they don’t know anything about what is going on during the pregnancy stage, or when the baby is born, they are not there. (IDI16)

The framing of the study by female Bukhali participants to their male partners also appears to have contributed to this gendered view of the research and topic of study:

She didn’t tell me the whole information, like, it’s about the girl when you are pregnant, it’s about your [women’s] body parts, she didn’t tell me a lot. (IDI4)

This highlights a possible limitation of *Bukhali*, which produced material about women’s heath during pregnancy and infant development. Producing material that is gender neutral or father inclusive, and providing it to the women participants, could provide a channel to open communication between partners and raise interest in the fathers.

On the other hand, some participants reflected that they became interested in the research on realizing that the female partners’ social situation, including their relationship and social support, might be a topic of conversation. This seemed to make the research more directly relevant to the fathers.

Then she tells me, “Okay they are going to ask me questions about this, they are going to ask me questions about my family, my background. They are going to ask me questions about my relationship”, then I’ll say, all respect, ja, that is when I became interested, it involves my relationship. (IDI10)

#### Resistance to sharing personal matters

In seeming contradiction to the above point, fathers described a tendency amongst their peers of not wanting to talk about their lives or discuss personal matters. This seemed to stem from a sense of pride and men not ‘normally’ sharing or showing emotions: ‘they stay bottled up’. This reluctance seemed to extend to engagement with *Bukhali*, due to the notion that participation entails questions about personal aspects of their lives.

I’d say most men don’t feel free speaking about their lives or their experiences… I would say maybe it is a sense of pride: Why should another guy know what is happening in my life? But it is, actually, sometimes it is quite good to speak to someone. (IDI18)

Most guys are shy, they don’t want to talk. (IDI15)

Another reason for the reluctance to share personal information was thought to be to avoid accountability or judgement for risky behaviours, such as infidelity and unsafe sex. Fathers felt that participation in the trial may result in confrontation around such actions and their possible health consequences.

Guys have a lot of things that they are hiding which maybe they know what they’re up to, so when they come here they know things will be revealed which they don’t want revealed….You know that most guys have many girlfriends on the side, so when these things come up they’ll think, “Eish, I slept with that girl without a condom” or “I did this and that so I’m not going there because what if they test me?” (IDI13)

#### Lack of time and (financial) benefits

Time ‘wasted’ and the need to prioritize time spent on often financially or materially beneficial pursuits were other reported factors discouraging fathers from participating.

Some are just lazy to get up and go out there and be part of something… there are no benefits, it is not like they are going to give me a job or anything, you are just going to take my time. Just for free…Most people want their time not go to waste, especially the guys, because some of them are busy trying to hustle out there, get money, and then when something like this comes up we just don’t feel interested. (IDI2)

### Fathers’ recommendations for improving engagement

#### Increasing awareness

Fathers’ main suggestion to improve men’s engagement was to increase awareness of the research through advertising and information provision. Opinions on the best way to achieve this varied, however. Some suggested face-to-face handing out informative pamphlets or flyers at public places, such as areas of public transportation, malls, or clinics. Others suggested using social media and garnering attention through marketing techniques.

You can maybe make flyers and give away flyers at the taxi ranks so that people… you must also go out there, where they are. (IDI15)

Social media maybe, mini events, just to get people hyped up, maybe do pop-ups at the malls. (IDI9)

There needs to be programs in the township for marketing the study; so that people can know that, ‘we are so and so and this is where we are from’; then you must educate the community to say, “this is how it is’. (IDI8)

Some fathers felt that having in-person conversations with men would be more effective than using media or pamphlets to spread information. They also suggested that the research team could leverage their female participants to disseminate information and understanding around the study, implying that female participants be made responsible for reaching and teaching their male partners.

You can do workshops, you can go to clinics, have a campaign… if you give away pamphlets people will never be engaged, but if you talk to them, engage with them, they will be involved more. (IDI12)

There’s no other way than obviously it’s our partners that need to encourage us to… they need to make us understand, because if they attend the study, obviously they have knowledge. (IDI1)

#### Creating a safe space

Fathers additionally suggested creating an environment where men feel comfortable and safe to talk about their issues, either by starting the interaction in their homes or by creating a more male-centred environment.

Even though they don’t want to come here so maybe you can go to them at least; and then when they are comfortable they can come here. (IDI16)

There’s a meeting, but it’s a man’s meeting, and if people have problems there can be a day specifically set aside whereby there will be a one-on-one, where we sit around together. (IDI5)

In sum, the male partners of *Bukhali* participants offer several themes for reflection under motivators and barriers for men’s engagement in reproductive research, as well as providing recommendations for their improved engagement.

## DISCUSSION

In this study, we asked the question: what are the motivators, barriers, and suggested improvements for men’s engagement with reproductive and lifecourse health research in SA? In the context of the *Bukhali* trial, we identified various motivators and obstacles to men’s engagement with the research, as well as several participant-directed recommendations for improving this engagement. These findings contribute to the currently limited literature on men’s engagement in lifecourse and reproductive research.

Firstly, our findings support the idea that men do feel motivated to participate in research that addresses reproductive health, which further challenges the notion that South African men simply lack the will or interest to engage (Venables and Stadler 2012, Grace *et al*. 2019, Nkwonta and Messias 2019, Draper *et al*. 2023b). Their motivation to participate appeared to be stimulated by multiple overlapping interests, regarding the self, the partner relationship, and the child. Regarding self-interest, and as noted in other trials in SA (Stadler *et al*. 2008, Cousins and Reynolds 2014), participants wanted to be involved on the basis that they might receive benefits. This included the same perceived rewards as their female partners, such as acquiring knowledge about pregnancy and general health literacy resources, and the opportunity for health checks. This finding implies a gap in accessible information on such topics for men.

The partner relationship was also a significant motivating factor for engaging with reproductive and lifecourse research, both as a natural result, and explicit expression, of their commitment and support to their partner. Prior research has shown partner support to be a major motivator for, and subsequent benefit of, participating in reproductive health research (Venables and Stadler 2012, Harlow *et al*. 2020, Drysdale *et al*. 2022). Our own prior work on relationship dynamics with these same participants explicates this fully (Draper *et al*. 2023a). Prioritizing the partner relationship may also indicate a shifting perception of fatherhood and masculinity towards one that is more equitable (Morrell *et al*. 2016, Draper *et al*. 2023a), e.g. where IDI11 says: ‘she mustn’t be the mother alone … as if she’s the only parent for the child’. However, male partner involvement in antenatal and wider reproductive services and research is not the norm in South African public health systems (Drysdale *et al*. 2021). While recruitment messaging could engage men by emphasizing the partner relationship, it should avoid reinforcing the reductionist concept of ‘men-as-support’ only, which risks framing men as secondary and auxiliary in reproductive research (Daniels 2006, Almeling and Waggoner 2013), and may only may reinforce gender inequality and harmful gender norms (Fleming *et al*. 2014).

Male partners also had implicitly expressed child-orientated motivators for engaging in reproductive research. Our participants tended to speak about this motivation as connected to their own identities or their relationships, instead of being purely about the child’s interest or bond, e.g. through desiring to become a ‘better father’ (IDI17) and ‘better person’ (IDI17). Prior research has foregrounded child/foetal bonding in men’s engagement with reproductive health research, for example, regarding antenatal ultrasounds, (Drysdale *et al*. 2022). Our male participants highly valued being present for the foetal ultrasound offered as part of the *Bukhali* trial. This appeared, however, to be primarily insofar as it enhanced *partner* bonding and being indicative of the trial’s legitimacy, with the foetus or future child not being explicitly mentioned by the male partners. Our research thus appears to show that while men do have child-related motivations to engage lifecourse, the benefits to the self and the partner relationship are stronger motivators. While the reason for this is not known, it could perhaps reflect gendered ideas of childcare [where the mother is thought to more directly affect the child’s wellbeing (Daniels 2006)] and feelings that their presence in antenatal activities may only influence the wellbeing of their child *via* the support to their partner.

Barriers to engaging in research appear to centre around gendered expectations and masculinity norms, as well as misconceptions, unawareness, and mistrust of research. While gender and masculinity norms around reproduction and parenting may be shifting towards those that are more equitable (Ratele *et al*. 2012, Makusha and Richter 2014, Draper *et al*. 2023a), we found that gender norms still represent a significant barrier for men’s engagement with, and access to, reproductive services and research (Venables and Stadler 2012, Grace *et al*. 2019, Watson *et al*. 2023). While gaining knowledge was a motivating factor for our participants, many felt that this could be barrier for men generally. Men would first have to be willing to admit ignorance and potentially answer personal questions that they do not know the answers to. Reluctance here appears to stem from embarrassment and pride, which some of our participants felt they had to actively put aside in order to engage. Secondly, reluctance to gain knowledge related to reproduction and children appears to stem from the perception that these are ‘women’s issues’ and are ‘private’ (IDI9). A notable example was IDI9’s covert reading of his partner’s trial materials, showing that even when men want to engage in gender equitable ways, they do not feel it appropriate or acceptable for them to do so as dictated by gender norms (Hay *et al*. 2019, Heise *et al*. 2019, Weber *et al*. 2019, Watson *et al*. 2023).

These gender norms in health and reproduction are further reinforced by the real or perceived nature of the (biomedical) reproductive research space, and the public health service space more generally, as being women-dominated (Venables and Stadler 2012, Almeling and Waggoner 2013, Grace *et al*. 2019, OECD 2019). In SA, 80% of healthcare professionals are nurses, 90% of which are women (Posel and Casale 2019). As patients, women of reproductive age also engage with health services more frequently than men, with potential reasons being the focus on women and children in HIV services, women’s increased contact with health services during antenatal and obstetric care, and their perceived responsibility for child and family health (Nkuoh *et al*. 2010, Venables and Stadler 2012, Nkomo 2015). South African men, too, are subject to masculinity norms, such as being custodians of tradition. The potential result is a favouring of traditional over biomedical healthcare (Venables and Stadler 2012)—an occurrence that is also related to a mistrust of biomedicine. Masculinity norms also demand that men are strong and powerful; the hospital, however, is considered a ‘place for weak people’ (IDI13), antithetical to the masculine ideal. Finally, masculinity hegemonies can also promote risky and unsafe sexual practices (Parker *et al*. 2007), and men do not want these behaviours scrutinized or judged (Venables and Stadler 2012). These factors together implicitly reinforce that men do not, or should not, engage with health research and services.

Lastly, the gendered expectation for South African men to be the economic provider for their partner(s) and children, and the pressure to do so, is immense and has been noted as a barrier to men’s engagement in reproductive research (Richter and Morrell 2006, Mfecane 2008, Venables and Stadler 2012, Watson *et al*. 2023, Draper *et al*. 2023a). Our participants expressed the same barrier. Often the pressure to provide comes with increased judgement around how men spend their time, with the expectation that it should entail actively working or job-seeking to the exclusion of other activities, including research (Venables and Stadler 2012). SA has the highest rate of unemployment in Africa and globally (Cowling 2023). A further concern is that those more affected by poverty may be under greater pressure to spend their time earning money, thereby being less able to participate in, and reap the benefits of, (reproductive) health research, resulting in only greater inequality.

A final prominent barrier to men’s engagement according to our participants is mistrust in health-related research, including the broader clinical and hospital-based setting. Suspicion of biomedical and reproductive health services, particularly amongst Black South Africans, may arise from a significant precedent of breaches in medical ethics. This includes Apartheid-era Black ‘population control’ projects (Fassin 2015), which endure in public consciousness. More recently, the coronavirus disease 2019 (COVID-19) pandemic may have further exacerbated South Africans’ mistrust in biomedicine and state-run healthcare through increasing state corruption, poor communication, and inconsistency in authoritarian interventions (Moll 2021, Silubonde *et al*. 2023). It was against this backdrop that the *Bukhali* participants and fathers were recruited and interviewed.

Fathers did indeed communicate initial feelings of suspicion, scepticism, and fear around this research. Mistrust ranged from concerns around being ‘scammed’, linguistic issues around the word ‘study’ (associated with reduced competency), and perceived danger within the hospital environment. This last concern included potential direct harm, e.g. through stealing organs and being viewed as a ‘place of death’ (IDI13), as well as indirect social and psychological consequences and stigma around attendance or particular diagnoses, such as HIV (Mfecane 2008, Venables and Stadler 2012). Mistrust is also significantly gendered; e.g. Venables and Stadler (2012) found in their HIV microbicide research that male partners tended to be more mistrusting than women participants, with suspicions around adequate testing and safety. They found that engaging the male and female partner dyad simultaneously in the research improved partner communication and decreased trial-related concerns and mistrust.

Our participants made two broad recommendations to overcome men’s barriers to engagement. Firstly, and especially relevant to the gendered barriers for men in the public health system (Drysdale *et al*. 2021), they recommended being engaged in spaces in which they feel comfortable and safe, such as outside the clinic, or in men-only meetings. Fathers did not recommend gender-inclusive or gender-neutral spaces, which this has been suggested in other research (Mohlala *et al*. 2012, Venables and Stadler 2012), especially as having a separate research site specifically for men may be infeasible. Care should also be taken to prevent siloing health according to gender, thereby reinforcing gendered spaces and hegemonies. Having one gender-inclusive space, however, would necessitate ensuring safeguards were in place for any female participants who may feel that their partner’s involvement would be detrimental for them (Venables and Stadler 2012). Gender-inclusive spaces should therefore employ strategies that prevent women from feeling disempowered or discriminated against. A gender-inclusive space in research may include employing more men, alongside women, in reproductive research and broader public health service settings (Grace *et al*. 2019). This was found to be effective in prior HIV and gender research, where male facilitators could challenge male participants’ and community members’ harmful masculinity norms by providing an alternative example of the facilitators’ own ‘successful’ masculinity (Gibbs *et al*. 2020).

A lack of men’s engagement with health research and services (and conversely, a lack of their engagement with men) circularly leads to a lack of men’s awareness of *opportunities* for their engagement (Grace *et al*. 2019). In line with this, fathers highlighted the importance of increasing awareness and communication. There was, however, no consensus about the best way to increase awareness, with suggestions ranging from in-person marketing to generating social media ‘hype’. The latter strategy has proven effective in other Sub-Saharan African settings (Nkwonta and Messias 2019). Incorporating men’s own recommendations into demonstrably effective methods that promote gender equity has great potential for improving men’s engagement in reproductive and lifecourse research.

## CONCLUSION

We find that a lack of men’s engagement in reproductive research is not simply attributable to their own choice or lack of interest, but is instead due to more complex gendered expectations and structural barriers concerning both researchers and researched. Besides our participants’ recommendations and reproductive and lifecourse research ought to start from a position that recognizes that men want to engage, thereby countering the ‘notion that men are not expected to be interested and engaged [which] thus becomes a self-fulfilling prophecy’ (Grace *et al*. 2019, p. 1). While there is a distinction between engagement with reproductive *services* and reproductive *research*, we found significant overlap in the motivators, barriers, and recommendations for men’s engagement in both. Thus, findings from our research, both novel and already established, can be leveraged to promote men’s engagement in future research as well as reproductive health services. This will assist in achieving the goals of the South African National Integrated Men’s Health Strategy (National Department of Health South Africa 2020). Additionally, potential collaborations with SA’s gender advocacy and health groups (such as Sonke Gender Justice, Men as Partners, and Brothers for Life), whose aims include promoting gender justice and transforming health and parenting norms, may provide unique opportunities for recommendation implementation. Multidisciplinary and multisectoral collaboration with such groups would positively impact not only men and boys but also women and children’s health (as their outcomes have been shown to improve with men’s greater involvement) [Sanaati *et al*. 2018, Nkwonta and Messias 2019)] and the project of gender justice itself.

## Data Availability

Data can be made on reasonable request to the corresponding author.
